# SV40 Large T Antigen Disrupts Embryogenesis of Canine and Porcine Somatic Cell Nuclear Transfer Embryo

**DOI:** 10.1186/s12575-017-0061-6

**Published:** 2017-10-18

**Authors:** Kiyoung Eun, Seon-Ung Hwang, Yeon Woo Jeong, Sunyoung Seo, Seon Yong Lee, Woo Suk Hwang, Sang-Hwan Hyun, Hyunggee Kim

**Affiliations:** 1 Department of Biotechnology, College of Life Sciences and Biotechnology, Korea University, 145 Anam-ro, Seongbuk-gu, Seoul, 02841 Republic of Korea; 2 Laboratory of Veterinary Embryology and Biotechnology, College of Veterinary Medicine, Institute of Stem Cell & Regenerative Medicine, Chungbuk National University, 52 Naesudong-ro, Seowon-gu, Cheongju, 28644 Republic of Korea; 3 Sooam Biotech Research Foundation, San 43-41 Oryu-dong, Guro-gu, Seoul, Republic of Korea; 4 Department of Medical Engineering, College of Medicine, Korea University, Seoul, Republic of Korea

**Keywords:** Simian virus 40 large T antigen, Immortalization, Somatic cell nuclear transfer, Embryogenesis, Canine fibroblast, Porcine fibroblast

## Abstract

**Background:**

Somatic cell nuclear transfer (SCNT) is a useful biotechnological tool for transgenic animal production using genetically modified somatic cells (GMSCs). However, there are several limitations preventing successful transgenic animal generation by SCNT, such as obtaining proper somatic donor cells with a sufficiently long life span and proliferative capacity for generating GMSCs. Here, we established simian virus 40 large T antigen (SV40LT)-mediated lifespan-extended canine fibroblast cells (SV40LT-K9 cells) and evaluated their potential as nuclei donors for SCNT, based on cellular integrity and SCNT embryo development.

**Results:**

SV40LT did not cause canine cell transformation, based on cell morphology and proliferation rate. No anchorage-independent growth in vitro and tumorigenicity in vivo were observed. After SCNT with SV40LT-K9 cells, embryos were transferred into surrogate dogs. All dogs failed to become pregnant. Most embryos did not proceed past the 8-cell stage and only one surrogate showed an implantation trace in its oviduct, indicating that the cells rarely developed into blastocysts. Because of the absence of an in vitro maturation method for canine embryos, we performed identical experiments using porcine fibroblast cells. Similarly, SV40LT did not transform porcine fibroblast cells (SV40LT-Pig cells). During in vitro development of SV40LT-Pig cell-driven SCNT embryos, their blastocyst formation rate was clearly lower than those of normal cells. Karyotyping analysis revealed that both SV40LT-K9 and SV40LT-Pig cells had aberrant chromosomal statuses.

**Conclusions:**

Although lifespan-extended canine and porcine cells via SV40LT exhibit no apparent transforming changes, they are inappropriate for use as nuclei donors for SCNT because of their aneuploidy.

## Background

Somatic cell nuclear transfer (SCNT) is an animal cloning technique that involves implanting a somatic cell into an enucleated oocyte [1]. This method has been remarkably improved and applied in various mammals, including cattle, goat, pig, horse, cat, and dog, since the successful birth of ‘Dolly’ the sheep in 1996 [2–8]. SCNT, compared with germline transmission, is considered a useful method for producing transgenic (TG) animals simply by modifying DNA of interest in the genome of somatic donor cells prior to nuclear transfer. The advantages of SCNT are clear in the generation of TG large animals with a long reproduction cycle because a long time is required to verify TG models at the individual level. Recently, because of technical advances in SCNT, large animals such as pigs have been used as human disease models rather than rodents in the translational research field [9].

However, primary animal somatic cells, which are used as nucleus donors, show a finite cellular lifespan in vitro; at the end of this lifespan, the cell enters a state of irreversible replicative senescence [10]. This phenomenon was first reported by Hayflick and found to be related to the “end replication problem,” which leads to telomere shortening with each cell division [11, 12]. Establishing TG donor cells has been challenging because these cells show senescence during modification of the gene of interest. Immortalization or extension of cellular lifespan is considered a plausible approach for overcoming these problems if genetically modified somatic cells (GMSCs) are normally reprogrammed during embryogenesis and further developed without cellular transformation.

Simian virus 40 large T (SV40LT) is a viral protein from simian virus 40, a member of *Polyomaviridae*. SV40LT is known to interact with various host proteins, including p53 and retinoblastoma, which inhibit cell proliferation [13]. A previous study showed that SV40LT immortalizes primary cells such as mouse embryonic fibroblast cells [14]. Additionally, numerous studies suggested that ectopic expression of SV40LT enhances induced pluripotent stem cell generation efficiency or even replaces Klf4 and c-Myc among four Yamanaka reprograming factors [15, 16]. Thus, SV40LT does not disturb cellular reprogramming or dedifferentiation from specific somatic cells up to the pluripotent embryo stage.

We attempted to generate TG canine cancer models by SCNT, as canine cancers are comparable to human cancers in terms of histological features, molecular profiles, drug responses, and therapy resistance [17]. However, the lifespan of canine fetal fibroblast cells is too short to construct the TG donor cell lines necessary for TG canine modeling by SCNT. To overcome this problem, we established lifespan-extended canine fetal fibroblast cells via SV40LT and evaluated these cells as nuclei donor cells for SCNT.

## Methods

### Cell Culture

Ear tissues separated from stillborn pigs (*Sus scrofa domesticus*) and dogs (*Canis lupus familiaris*) were washed in Dulbecco’s phosphate-buffered saline-positive containing 1% (*v*/v) antibiotic-antimycotic (Gibco, Grand Island, NY, USA). Ear tissues were sterilized in 70% ethanol and washed with Dulbecco’s phosphate-buffered saline-positive. Skin tissues were separated from sterilized ear tissue and chopped finely using a blade. Chopped skin tissues were incubated with 0.25% trypsin for 2 h and then added to the cell culture medium [Dulbecco’s modified Eagle’s medium (DMEM; Gibco) containing 10% (*v*/v) fetal bovine serum (FBS; Gibco) and 2% (v/v) antibiotic-antimycotic (Gibco)] to neutralize the trypsin. Trypsinized tissues were cultured in a 100-mm culture dish and adherent cells were subcultured. The incubation conditions for primary culture were 37 °C and 5% CO_2_ in a humid incubator (Astec, Fukuoka, Japan). Dog and pig fetal fibroblast cells were maintained in DMEM supplemented with 10% FBS, 1% Glutamax (Gibco), 1% non-essential amino acids (Gibco), 1% antibiotic-antimycotic, and 0.1% 2-mercaptoethanol (Gibco).

### Lentiviral Vector Construction and Lentivirus Production

To prepare the lentiviral vector pLL-CMV-SV40LT-blast, the SV40LT gene was digested with two restriction enzymes, *Sal*I (blunted by Klenow fragment) and *SnaB*1 from pBabe-SV40LT-puro. Next, the insert DNA was ligated into the *Sma*I site of the pLL-CMV-Multi Cloning Sites-blast vector. To produce the lentivirus, 293FT cells were plated onto 100-mm plates in complete DMEM containing 10% FBS and 2 mM L-glutamine (Lonza, Basel, Switzerland) and allowed to adhere for 12 h. Transfection was performed as per the manufacturer’s instructions with 4 μg pLL-CMV-SV40LT-blast vector, 4 μg 3rd-generation packaging plasmids, and 24 μL Polyexpress agent (Excellgen, Inc. Rockville, MD, USA) in serum-free DMEM medium (Invitrogen, Carlsbad, CA, USA). Six hours after transfection, the cells were washed with PBS, and then mixed with fresh complete DMEM containing L-glutamine and 10% FBS and incubated for an additional 42 h. Supernatant medium containing lentivirus was collected 48 h after transfection and filtered through a 0.45-μm syringe filter. Each lentivirus was concentrated using a Lenti-X Concentrator (Clontech Laboratories, Inc., Mountain View, CA, USA) according to the manufacturer’s instructions.

### Establishment of Lifespan-Extended Fibroblasts

Dog and Yucatan miniature pig fibroblast cells were infected with pLL-CMV-SV40LT-blast lentivirus vector, using polybrene (6 μg/mL; Sigma-Aldrich, St. Louis, MO, USA). Three days after infection, cells were selected by blasticidin treatment (2 μg/mL; Clontech Laboratories, Inc.) for 5 days.

### Western Blot Analysis

Whole cell extracts were prepared using RIPA lysis buffer (150 mM NaCl, 1% NP-40, 0.1% SDS, and 50 mM Tris [pH 7.4]) containing 1 mM β-glycerophosphate, 2.5 mM sodium pyrophosphate, 1 mM NaF, 1 mM Na_3_VO_4_, and protease inhibitor cocktail (Roche, Basel, Switzerland). Protein levels were quantified using Bradford assay reagent (Bio-Rad, Hercules, CA, USA) according to the manufacturer’s instructions. Proteins were separated by SDS-PAGE and transferred to polyvinylidene difluoride membranes (Pal Corporation, Bronx, NY, USA) according to standard protocols. Membranes were immunoblotted with antibodies against c-H-RAS (Calbiochem, Darmstadt, Germany) and SV40LT antigen (Santa Cruz Biotechnology, Inc., Santa Cruz, CA, USA) in 3% bovine serum albumin Tris-buffered saline containing Tween-20 buffer. After primary antibody incubation, membranes were probed with horseradish peroxidase-conjugated goat anti-mouse IgG (Pierce Biotechnology, Inc., Rockford, IL, USA) or horseradish peroxidase-conjugated goat anti-rabbit IgG (Pierce Biotechnology, Inc.) secondary antibody. β-Actin was used as a loading control.

### Soft Agar Assay

A 1.5-mL lower layer of 0.72% agar in DMEM containing 10% FBS was placed in each well of a 6-well plate and allowed to solidify at room temperature (18–20 °C). Cells were suspended in a plating layer (1.5 mL) of 0.28% agar in DMEM containing 10% FBS. The agar was allowed to solidify at room temperature and incubated at 37 °C in a humidified CO_2_ incubator. To prevent the agar from drying, 500 μL DMEM containing 10% FBS was added to each well every 4–5 days.

### Subcutaneous Implantations for In Vivo Tumorigenesis Assay

All mouse experiments were approved by the animal care committee of Korea University and carried out in accordance with ethical standards of government and institutional guidelines and regulations. To establish subcutaneous xenograft models, 2 × 10^6^ cells of each cell line in PBS were mixed with Matrigel (Invitrogen) at a ratio of 50% and then subcutaneously transplanted into 5–6-week-old BALB/c nu/nu mice (Orient Bio, Inc., Seongnam, Korea). Tumor sizes were calculated using the following formula: tumor volume (mm^3^) = longest diameter of tumor (mm) × shortest diameter of tumor (mm) ^2^/2. The mice were sacrificed if unexpectedly found to be moribund or they showed rapid body weight loss (over 20% of body weight), hunched posture, lethargy or persistent recumbency, or a tumor estimated to be more than 10% of the body weight. Mice with tumors exceeding 1000 mm^3^ were sacrificed. Mice without tumors were maintained for up to 6 months, and then sacrificed by carbon dioxide asphyxiation in accordance with government and institutional guidelines and regulations.

### Canine Cloning

The estrous stage of female dogs (Hyundae Kennel, Seoul, Korea) was examined weekly by observing for vulval bleeding to detect the onset of the heat period. During heat, 2-mL blood samples were collected daily by cephalic venipuncture and serum P4 levels of blood samples were measured by electrochemiluminescence immunoassay (Cobas e411, Roche Diagnostics, Mannheim, Germany). Oocytes were retrieved surgically under general anesthesia after ovulation as reported previously [18]. After retrieval, metaphase II (MII) oocytes were stripped of cumulus cells and enucleated by squeezing out the first polar body and MII plate with a small amount of surrounding cytoplasm using a glass pipette. Oocytes were pre-stained with 5 mg/mL bisbenzimide (Hoechst 33,342; Sigma-Aldrich) to visualize the presence of nuclei indicating the enucleation process. Using a fine pipette, a trypsinized fetal fibroblast with a smooth cell surface was transferred into the perivitelline space of an enucleated oocyte. Next, the couplets were transferred to a chamber with two electrodes and covered with mannitol solution. The couplets were fused with two DC pulses using a BTX Electro-Cell Manipulator 2001 (BTX Inc., San Diego, CA, USA). As soon as fusion and activation were complete, all reconstructed embryos were loaded into a tomcat catheter (Severeign, Sherwood Medical, St. Louis, MO, USA) with a minimum volume of media and gently transferred into the distal position of the oviduct without insufflating air. Real-time ultrasonography was performed on the pregnant dog 25–30 days after embryo transfer and repeated every 7 days. The sizes and shapes of the chorionic cavities and presence of an embryonic or fetal heartbeat were examined to identify embryonic or fetal death.

### Embryo Collection after Dog Embryo Transfer

Two different methods were employed to collect the transferred embryos. The first method, ovariohysterectomy, involves flushing the reproductive tract by excision. Ovariohysterectomy was performed under general anesthesia on day 8 after embryo transfer. Under aseptic conditions, the reproductive tract was exposed through a midventral incision. Before excision, both uterine horns were ligated at the transition to the utero-tubal junction, 1 cm caudal to the ovarian bursa, to avoid artefacts in embryo location within the oviduct, including the utero-tubal junction and cranial uterine horn. Another ligation was fixed at the entrance of the uterine body to prevent embryo loss. Immediately after excision, the genital tract was immersed in physiological saline solution at 37 °C. For each of the three locations, all embryos were recovered. Their developmental stages were determined and assessed under a stereoscope at 900× magnification. The second method used was the Foley balloon catheter method, which involves flushing the reproductive tracts using a balloon tip catheter without excision. Uterine and oviduct flushing was performed under general anesthesia at 7–9 days after embryo transfer. Following surgical exposure of the reproductive tract, a needle puncture was made in the uterine body with a fine needle. After removing the needle, a balloon catheter was immediately placed in the uterine body caudal to the bifurcation of the uterus. After ligating the Foley balloon catheter into the cervix and inflating the balloon, the upper part of the designated segment of the uterus was ligated to prevent any backflow and the needle was inserted, allowing 10 mL TCM 199 (supplemented with HEPES flushing medium) to flow through three or four times. After collection, the perforation was closed with a single stitch.

### Pig Oocyte Collection and In Vitro Maturation (IVM)

Porcine ovaries obtained from a slaughterhouse were delivered to the laboratory in a thermos containing 0.9% saline and at 36 °C. Within 2 h, 3–6-mm ovarian follicles were aspirated using a 10-mL syringe with an 18-gauge needle. The obtained porcine follicular fluid was centrifuged at 500×*g* for 30 min at 4 °C, and then filtered through 0.45-μm filters. Cumulus-oocyte complexes (COCs) were washed using TLH-PVA medium [HEPES-buffered Tyrode’s medium (TLH) containing 0.05% (*w*/*v*) polyvinyl alcohol (PVA)], and then co-cultured with 50–60 COCs per well in a 4-well dish (Nunc, Roskilde, Denmark) containing 500 μL of IVM medium. IVM was performed in IVM medium containing 10 IU/mL equine chorionic gonadotropin and 10 IU/mL human chorionic gonadotropin for 22 h, and then the cells were moved to hormone-free IVM medium and cultured for 18 h. The composition of IVM medium was TCM199 (Gibco), 0.6 mM cysteine, 0.91 mM sodium pyruvate, 10 ng/mL epidermal growth factor, 75 μg/mL kanamycin, 1 μg/mL insulin, and 10% (*v*/v) porcine follicular fluid. The incubation conditions for IVM were 39 °C and 5% CO_2_ in a humid incubator (Astec). Mature COCs were denuded by gently pipetting with 0.1% hyaluronidase, and then washed with TLH-PVA medium. MII oocytes obtained by this process were used for subsequent experiments.

### Somatic Cell Nuclear Transfer (SCNT) and In Vitro Culture (IVC)

After IVM, denuded oocytes at the MII stage were selected for enucleation. MII oocytes were washed three times in calcium-free TLH containing 0.2% bovine serum albumin and 5 μg/mL cytochalasin B (CB; Sigma-Aldrich). Washed MII oocytes were stained with 10 μg/mL Hoechst 33,342 dye (Sigma-Aldrich) for 5 min and then enucleation was performed in TLH-bovine serum albumin-CB drop using a micro-manipulator with a 16-mm glass injection pipette (Humagen, Charlottesville, VA, USA). After enucleation, a trypsinized single donor cell was picked up using a 16-mm glass injection pipette and injected into the perivitelline space of the enucleated oocyte. Injected oocytes were washed with 280 mM mannitol and then fused with two pulses of 180 V/mm DC for 60 μs in 260 mM mannitol solution containing 0.1 mM CaCl_2_ and 0.05 mM MgCl_2_ using a cell fusion generator (LF201; Nepa Gene, Chiba, Japan). After electrical fusion, the SCNT embryos were incubated in 6-dimethyl aminopurine containing 5 μg/mL CB in 30-μL drops of porcine zygote medium (PZM) for 4 h. SCNT embryos were transferred to a PZM drop for IVC. On the second day after fusion, embryo cleavage was evaluated (1-cell, 2-cell, 4-cell, 8-cell, and fragment) and transferred to a new PZM drop. On the 7th day after fusion, blastocyst (BL) formation was evaluated (early BL, expanded BL, and hatched BL). The incubation conditions for IVC were 39 °C and 5% CO_2_ in a humid incubator.

### Pig Embryo Transfer

Non-superovulated, naturally cycling Landrace × Duroc crossbreed gilts were used as surrogate mothers. First, pigs were injected with 1 mL ketamine (50 mg/mL; Yuhan, Seoul, Korea) and 3 mL xylezine (100 mg/mL; SF Inc., Ansan, Korea) in the ear vein for pre-anesthesia. Respiratory anesthesia was maintained using isoflurane liquid (Hana Pharm, Seoul, Korea). All surgical instruments were sterilized. Midventral laparotomy was performed to expose the reproductive organ. At 4 h post-activation, SCNT embryos (10–15 embryos per recipient) were transferred into an oviduct at the ampullary isthmic junction. Pregnancy was diagnosed on day 30 by ultrasonography.

### Karyotyping

Canine and porcine fibroblasts were karyotyped commercially by GenDix, Inc. (Seoul, Korea). Karyotyping results were analyzed using the ChIPS-Karyo program, a chromosome image processing system. Analysis data and all terms were represented using the International System for Human Cytogenomic Nomenclature 2016 (ISCN 2016).

### Statistical Analysis

All experiments were conducted more than three times. All data were analyzed by one-way analysis of variance followed by Duncan’s test using SPSS software (SPSS, Inc., Chicago, IL, USA) and the results are reported as the mean ± standard error of mean. Statistical differences were considered significant if the *p* value was less than 0.05.

## Results

### SV40LT Leads to Extension of Canine Fibroblast Cell Lifespan without Inducing Cancerous Properties

**Fig. 1:**
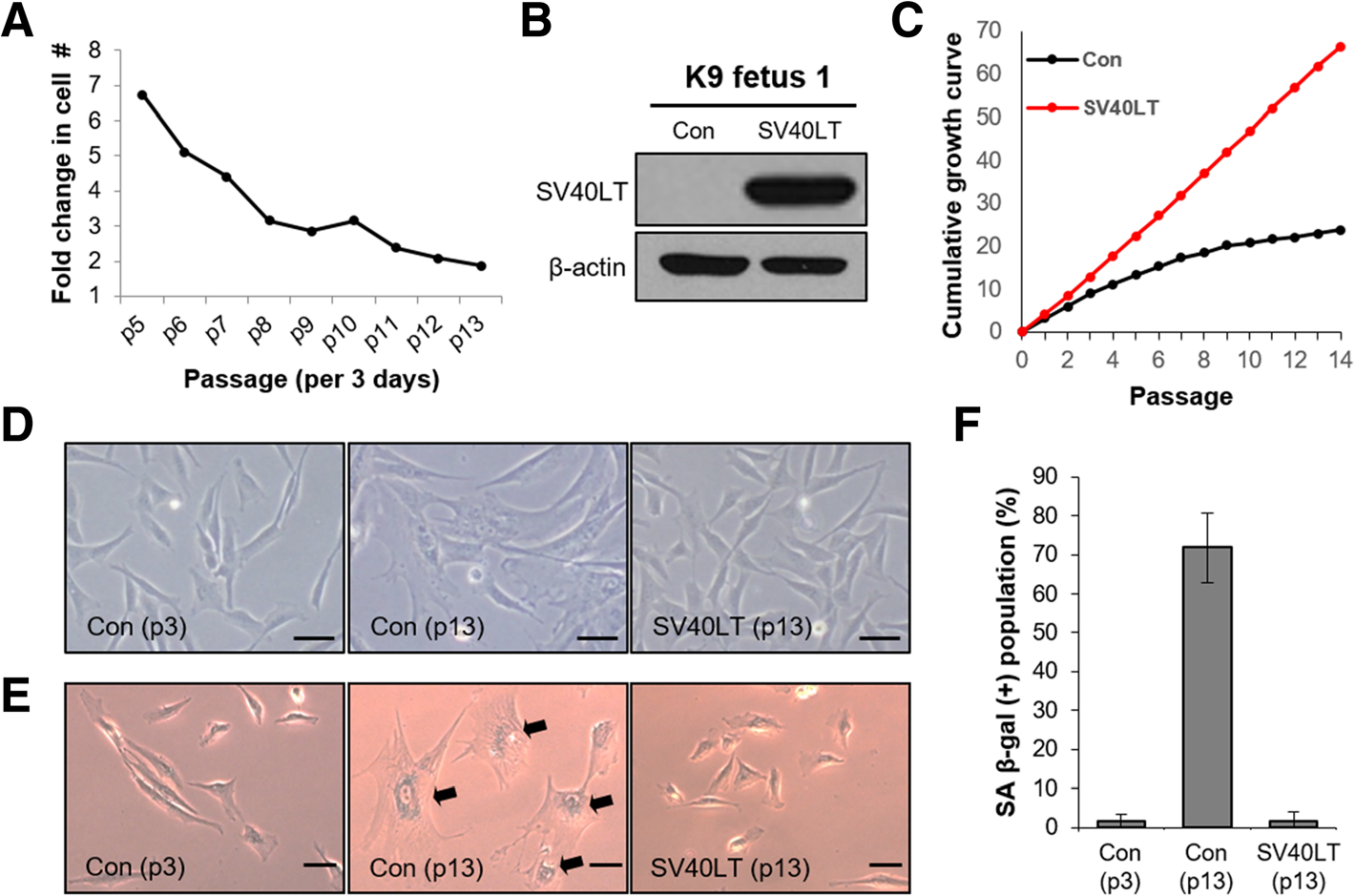
Immortalization of canine primary fibroblast cells via ectopic expression of SV40LT. **a** Cell growth rates (fold-changes) of different passages of K9 fetus 1 fibroblast cells were examined by counting 3 days after plating (1 × 10^5^). **b** Western blotting analysis showing expression of SV40LT in control K9 fetus 1 fibroblast cells and cells expressing SV40LT. β-Actin was used as a loading control. **c** Cumulative growth curves of control K9 fetus 1 fibroblast cells and cells expressing SV40LT. **d** Microscopic images showing cellular morphology of control K9 fetus 1 fibroblast cells (passages 3 and 13) and cells expressing SV40LT (passage 13 after antibiotic selection). Scale bars indicate 5 μm. **e** Senescence-associated β-galactosidase (SA-β-gal) stain assay of control (passages 3 and 13) and SV40LT-overexpressing K9 fetus 1 fibroblast cells (passage 13). Arrows indicate SA-β-gal-positive cells in passage 13 of control K9 fetus 1 fibroblast cells. Scale bars indicate 5 μm. **f** Quantitative analysis of SA-β-gal-positive cells presented in (E). P# indicates passage number of cells

Our primary fetal canine fibroblast line, K9 fetus 1, had a very short cellular lifespan, showing the senescence phenotype at approximately passages 5–7 (Fig. 1a). The growth of these cells was nearly halted after passage 13, with a marked increase in cell sizes and senescence-associated β-galactosidase (SA-β-gal) activity (Fig. 1d–f). To extend the life-span of these cells, we overexpressed SV40LT in K9 fetus 1 cells using a lentiviral vector (Fig. 1b). SV40LT overexpression led to continuous proliferation without a decrease in the growth rate, cell morphological changes, and SA-β-gal senescence phenotype (Fig. 1c–f). Taken together, these results indicate that SV40LT increases the lifespan of primary canine fibroblast cells.

**Fig. 2:**
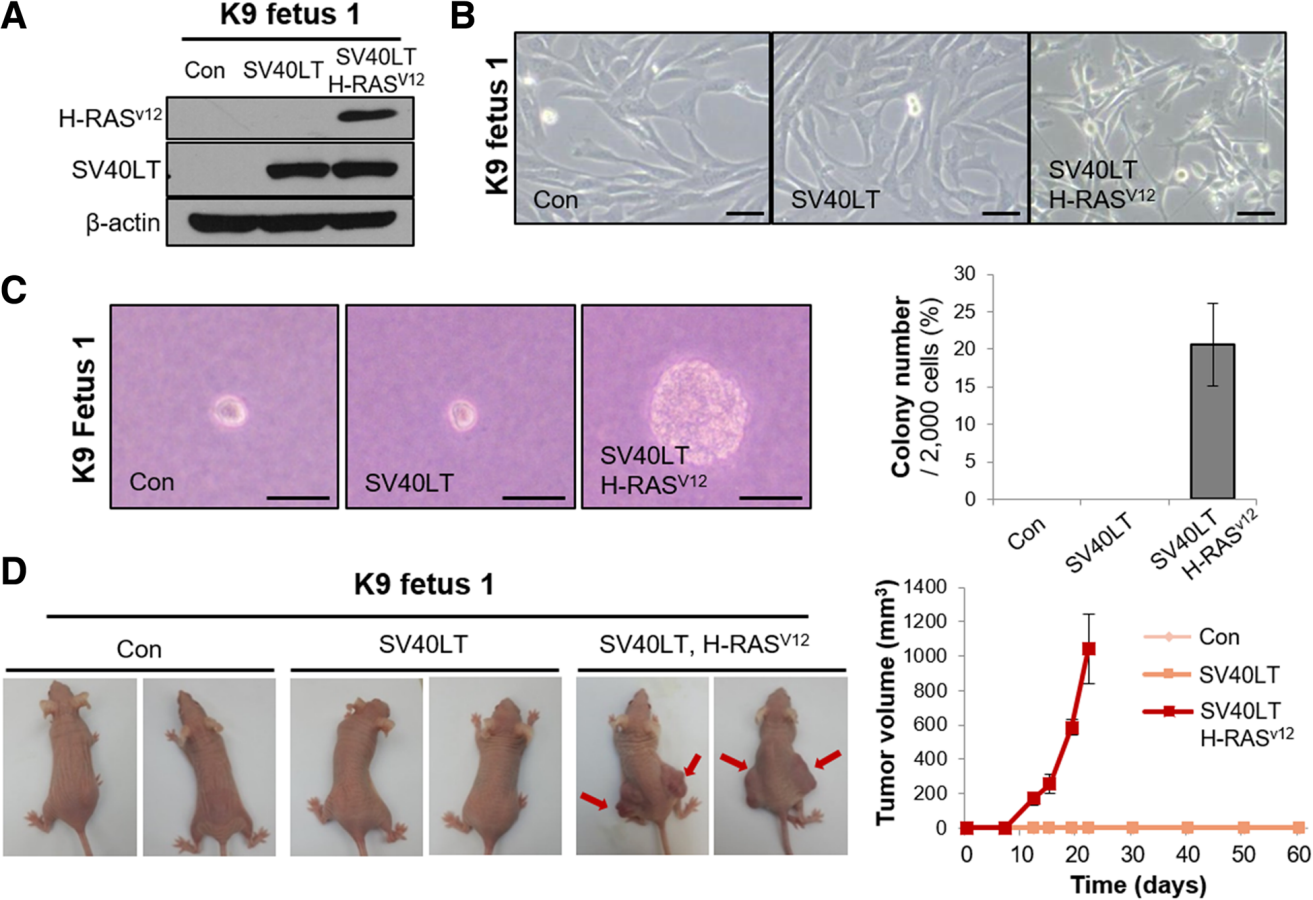
Non-transformed characteristics of SV40LT-mediated immortalized canine fibroblast cells. **a** Western blotting analysis showing expression of SV40LT or H-RASv12 in control K9 fetus 1 fibroblast cells, cells expressing SV40LT, and cells expressing SV40LT and H-RAS^V12^. β-Actin was used as a loading control. **b** Microscopic images showing cellular morphology of control K9 fetus 1 fibroblast cells, cells expressing SV40LT, and cells expressing SV40LT and H-RAS^V12^. Scale bars indicate 5 μm. **c** Microscopic images showing colonies formed under soft agar culture conditions (left photos). Quantitative analysis showing colony number of control K9 fetus 1 fibroblast cells, cells expressing SV40LT, and cells expressing SV40LT and H-RAS^V12^ (right graph). Scale bars indicate 3 μm. **d** Representative photos (left panel) showing immuno-deficient mice injected with control K9 fetus 1 fibroblast cells, cells expressing SV40LT, and cells expressing SV40LT and H-RAS^V12^. Arrows (red) indicate xenograft tumors derived from cells expressing SV40LT and H-RAS^V12^. Quantitative analysis showing in vivo tumorigenic potential of control K9 fetus 1 fibroblast cells, cells expressing SV40LT, and cells expressing SV40LT and H-RAS^V12^ (right graph)

It has been reported that SCNT embryos from malignant melanoma cells exhibit unsuccessful blastocyst development [19], indicating that some cancerous characteristics involving genetic or epigenetic status affect the reprogramming process. Because a previous study demonstrated that SV40LT enabled conversion of some types of normal cells into cancerous cells [20], we examined whether SV40LT-overexpressing K9 fetus 1 cells showed cancer cell properties by comparison with SV40LT-overexpressing K9 fetus 1 cells transduced with H-RAS^V12^, an oncogenic mutant of H-RAS (substitution of the 12th glycine to valine) (Fig. 2a). K9 fetus 1 cells expressing SV40LT alone showed no cellular morphological changes compared to control counterpart cells, whereas K9 fetus 1 cells expressing both SV40LT and H-RAS^V12^ showed relatively smaller, rounded, and refractive shapes by phase-contrast microscopy, which are typical characteristics of transformed cells (Fig. 2b). Control and SV40LT-overexpressing K9 fetus 1 cells did not show anchorage-independent growth, which are a feature of cancer cells in vitro, under soft agar culture conditions (Fig. 2c). However, there was a marked increase in the number of colonies of K9 fetus 1 cells expressing SV40LT and H-RAS^V12^ under the same culture conditions (Fig. 2c). All cells were subcutaneously transplanted into immuno-deficient nude mice to examine their in vivo tumorigenic potential. The results showed that control and K9 fetus 1 cells expressing SV40LT alone did not cause tumor formation for up to 6 months, whereas K9 fetus 1 cells expressing both SV40LT and H-RAS^V12^ caused tumor formation (Fig. 2d). These results suggest that SV40LT-mediated immortalized canine fibroblast cells were not transformed.

### Canine SCNT Embryos Derived from SV40LT-Expressing Fibroblast Cells Failed to Cause Pregnancy

The immortalized fibroblast cell used as a nucleus donor for SCNT does not disrupt normal embryonic development. Thus, we performed nuclear transfer (NT) using SV40LT-mediated immortalized canine fibroblast cells (SV40LT-K9 cells). We successfully fused 305 of 696 oocytes (43.5%) with SV40LT-K9 cells, which were transferred into 21 surrogates. However, no animals became pregnant (Table 1). In contrast, 109 putative embryos (of 121 oocytes; 90.1%) fused with normal fibroblasts were transferred into 7 surrogates, resulting in 3 healthy puppies (Table 1).

**Table 1:** Pregnancy outcomes of canine embryos derived from SCNT using control and SV40LT-expressing fibroblast cells

Donors	Nuclear transfer					Embryo transfer		
	No. of					No. of surrogate dogs	No. of pregnancies (%)^†^	
	Oocyte donor dogs	Oocyte subjected to NT	Oocyte fused & transferred (%)*	Sac observed (%)^**^	Offspring born (%)**			
							On Day 30	Full Term^‡^
Con-K9 cells	14	121	109 (90.1)^a^	3 (2.8)^a^	3 (2.8)^a^	7	3 (21.1)^a^	3 (21.1)^a^
SV40LT-K9 cells	72	696	305 (43.8)	0 (0.0)	0 (0.0)	21	0 (0.0)	0 (0.0)

^†^Pregnancy was detected by real-time ultrasonography. Pregnancy rate was calculated based on the number of pregnancies observed of the total number of surrogates transferred with reconstructed embryos (fused oocytes)

‡Full term pregnancy refers to pregnancy that successfully produces living offspring

*Fusion rate was calculated based on the number of fused oocytes of the total oocytes subjected to nuclear transfer

**The rate of sac observed and offspring born was calculated based on the total number of reconstructed embryos transferred

Values with superscript letter (a) within a column differ significantly (*p* < 0.05)

**Fig. 3:**
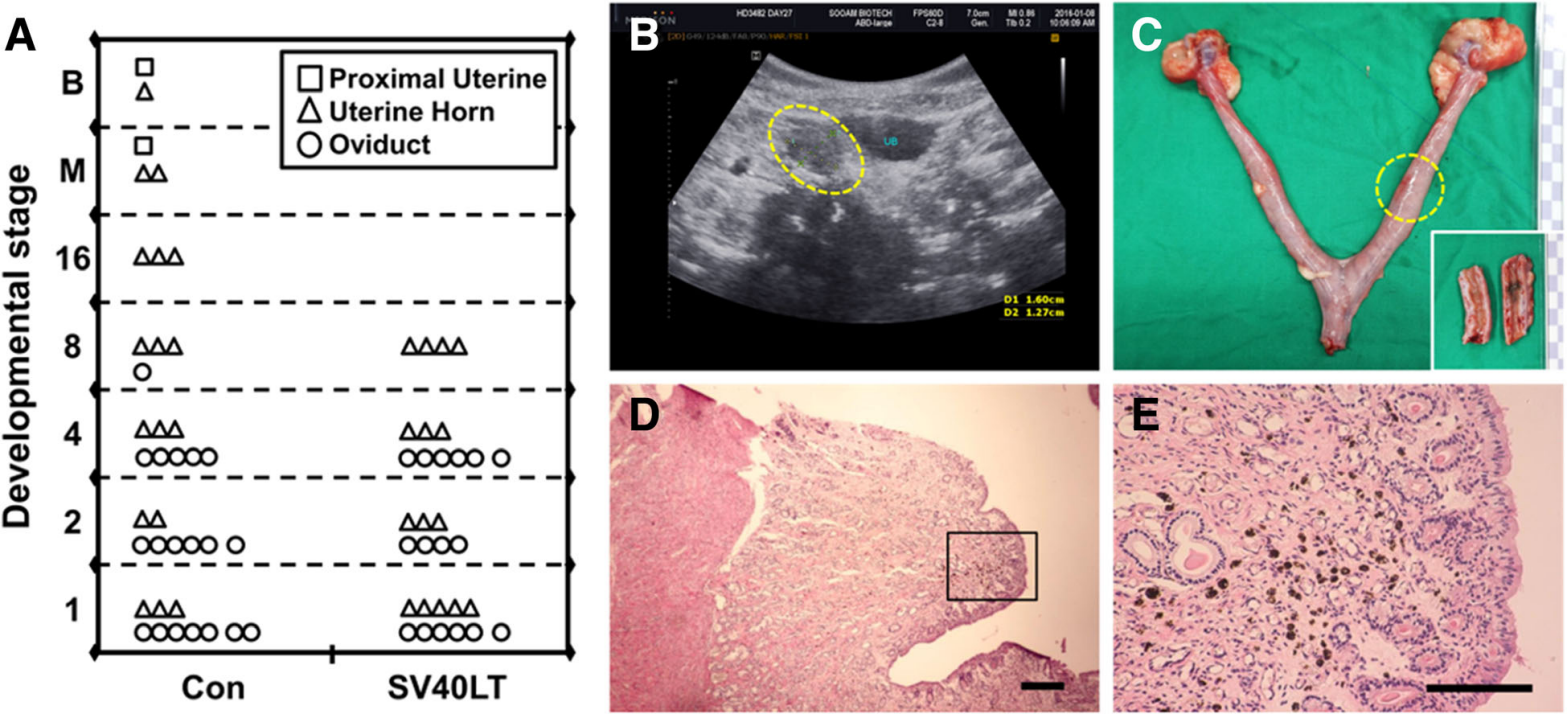
Developmental processes after embryo transfer of SV40LT-mediated immortalized canine fibroblast cells. **a** Localization of canine cloned embryos at different stages of development in the reproductive tracts. B, blastocyst; M, morula; n, total number of embryos recovered from each column or row. **b** Ultrasonographic image of thickened myometrium. A yellow dotted circle indicates well-demarcated uterine wall next to urinary bladder. **c** Retrieved female reproductive tract. A yellow dotted circle represents implantation scar and bisect uterus. **d** Histology of implantation site. Scale bar indicates 200 μm. **e** Multiple scattered hemosiderin spot. Embryos were recovered from surrogates after embryo transfer on day 8. Scale bar indicates 100 μm

Because the in vitro culture conditions for canine embryo have not been optimized, the developmental stages and localization of transferred embryos were analyzed histologically in the surrogate uterus and oviduct 8 days after embryo transfer. All embryos in the SV40LT-K9 cell group were in the oviducts (16 embryos of 31; 51.6%) and uterine horns (15 embryos of 31; 48.4%), and none proceeded to the 8-cell stage (Fig. 3a). In contrast, 5 embryos in the control fibroblast cell group developed to a morula and blastocyst stage progressed up to the nearby uterus (Fig. 3a). Interestingly, one surrogate exhibited a thickened myometrium by ultrasonographic examination on day 30 after embryo transfer (Fig. 3b). The surrogate uterus was retrieved and subjected to histological analysis, which revealed multiple scattered hemosiderin spots representing implantation traces (Fig. 3c–e). This indicates that rare cases of SCNT embryos derived from SV40LT-K9 cells progressed up to the blastocyst and implantation stage, although they failed to become pregnant. These data indicate that canine SCNT embryos derived from SV40LT-K9 rarely develop blastocysts after embryo transfer, resulting in pregnancy failure.

### Porcine Fetal Fibroblast Cells Expressing SV40LT Do Not Have Transformed Phenotypes

Unlike most mammalian species, canine SCNT embryos do not successfully develop into blastocysts in vitro. Therefore, it is difficult to determine which developmental processes are disrupted by SV40LT during SCNT embryogenesis. Alternatively, the pig is considered a suitable model because it is actively utilized for SCNT cloning and its embryo culture system in vitro has been well-established [21].

**Fig. 4:**
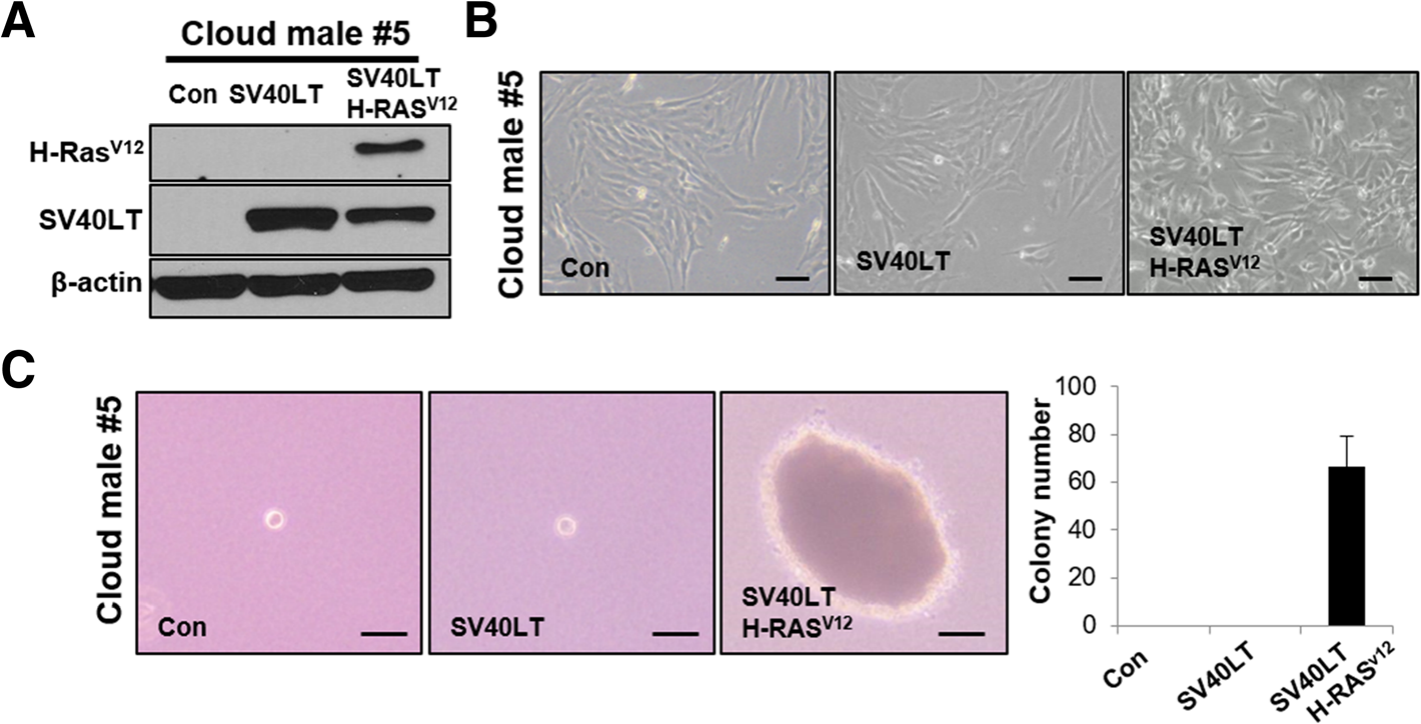
SV40LT did not transform porcine fetal fibroblast cells. **a** Western blotting analysis showing expression of SV40LT or H-RAS^V12^ in control porcine fetal fibroblast cells (Cloud male #5), cells expressing SV40LT, and cells expressing SV40LT and H-RAS^V12^. β-Actin was used as a loading control. **b** Microscopic images showing cellular morphology of control porcine fetal fibroblast cells, cells expressing SV40LT, and cells expressing SV40LT and H-RAS^V12^. Scale bars indicate 10 μm. **c** Microscopic images showing colonies formed under soft agar culture conditions (left photos). Quantitative analysis showing colony number of control porcine fetal fibroblast cells, cells expressing SV40LT, and cells expressing SV40LT and H-RAS^V12^ (right graph). Scale bars indicate 5 μm

Prior to SCNT, in vitro and in vivo experiments applied to canine fibroblast cells were identically conducted using porcine fetal fibroblast cells (Cloud male#5) overexpressing SV40LT alone or both SV40LT and H-RAS^V12^ (Fig. 4a). Similar to the results obtained for canine fibroblast cells, control and porcine fibroblast cells expressing SV40LT alone showed no morphological changes or anchorage-independent growth, while porcine fibroblast cells expressing both SV40LT and H-RAS^V12^ displayed marked changes in cell morphology and anchorage-independent growth (Fig. 4b and c), as observed for canine fibroblast cells expressing both SV40LT and H-RAS^V12^ (Fig. 2). However, *an* in vivo tumorigenesis assay showed that no porcine fibroblast cells developed tumors in immune-deficient nude mice (unpublished observations). Taken together, these results demonstrate that congruent to that in canine fibroblast cells, transformed traits were not detected in SV40LT-overexpressing porcine fibroblast cells.

### SV40LT Disrupts Blastocyst Development during Porcine Embryogenesis In Vitro

We next performed SCNT using three types of porcine fibroblast cells (control, cells overexpressing SV40LT alone, and cells overexpressing both SV40LT and H-RAS^V12^) as nucleus donors and evaluated the pattern of development for each embryo in vitro.

As shown in Table 2, the fusion rate of the SV40LT group was significantly lower than that of the SV40LT + H-Ras^V12^ group (61.0 ± 4.7 vs. 82.0 ± 4.7). However, there were no significant differences in fusion rates between the control group and SV40LT group (71.8 ± 6.1 vs. 61.0 ± 4.7) or control group and SV40LT + H-RAS^V12^ group (71.8 ± 6.1 vs. 82.0 ± 4.7). The total cleavage rates (2–8-cell) in the SV40LT group were significantly lower than those in the control group and SV40LT + H-RAS^V12^ group (48.6 ± 2.4 vs. 73.8 ± 4.0 and 48.6 ± 2.4 vs. 72.8 ± 8.2, respectively). However, this difference was not due to the SV40LT gene, as there was no significant difference between the control group and SV40LT + H-RAS^V12^ group (73.8 ± 4.0 vs. 72.8 ± 8.2). Remarkably, blastocyst formation rates in the SV40LT and SV40LT + H-RAS^V12^ groups were significantly (*p* < 0.05) lower than that in the control group (5.6 ± 1.8, 6.2 ± 1.8 and 19.5 ± 1.2, respectively). Interestingly, all developed blastocysts in the SV40LT and SV40LT + H-RAS^V12^ groups showed rough morphologies or unclearly extended inner cell mass structures (unpublished observations). These results suggest that consistently with the results for canine SCNT embryos, porcine SCNT embryos expressing the SV40LT transgene exhibited disrupted blastocyst development.

**Table 2:** Effects of SV40LT and H-RAS^V12^ genes in porcine blastocyst development of SCNT embryos

Donor cells	No. of	Fused (%)	No. (%)* of embryos developed to	
	oocytes		≥2-cell	Blastocyst
Con-Pig cells	250	169 (71.8 ± 6.1)^a b^	124 (73.8 ± 4.0)^a^	33 (19.5 ± 1.2)^a^
SV40LT-Pig cells	246	141 (61.0 ± 4.7)^a^	69 (48.6 ± 2.4)^b^	8 (5.6 ± 1.8)^b^
SV40LT + HrasV12-Pig cells	274	201 (82.0 ± 4.7)^b^	153 (72.8 ± 8.2)^a^	13 (6.2 ± 1.8)^b^

The cleavage rate (2–8-cell stage) was measured on day 2 and the blastocyst formation rate was evaluated on day 7 of culture

Values with different superscript letters (a, b, c) within a column differ significantly (*p* < 0.05)

Experiment was repeated three times

The data represent the means ± SEM

*Percentage of total fused oocytes

### Chromosomal Aberration Induced by SV40LT-Mediated Immortalization May Disrupt Blastocyst Development

**Fig. 5:**
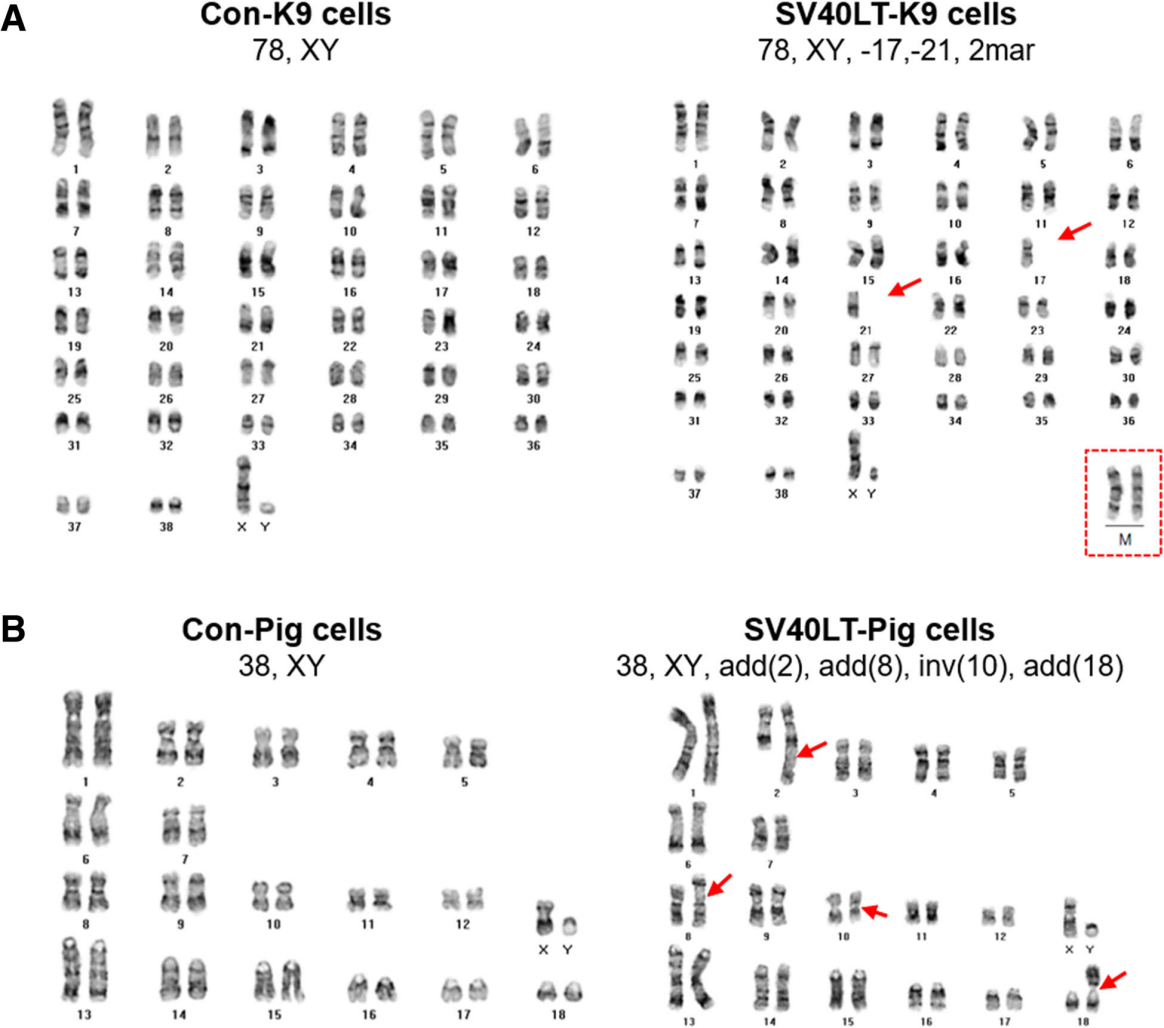
Aberrant karyotypes in both SV40LT-K9 cells and SV40LT-pig cells. **a** Karyotype analysis of Con-K9 cells and SV40LT-K9 cells. **b** Karyotype analysis of Con-Pig cells and SV40LT-Pig cells. Red arrows indicate abnormal chromosomes and red dotted box indicates additional unidentified marker chromosome. -, missing chromosome; mar, marker chromosome; add, additional material of unknown origin; inv., inversion

Previous studies suggested that blastocyst morphology and development are closely associated with the chromosomal status of the embryo [22–25]. To determine the reason for embryogenesis failure of SCNT embryos derived from SV40LT-mediated immortalized canine and porcine fibroblasts, we performed karyotype analysis to identify their chromosomal status. The results showed that both SV40LT-K9 cells and SV40LT-Pig cells contained chromosomal aberrations, whereas control cells had normal karyotypes (Fig. 5a and b). These results suggest that SV40LT-mediated immortalization leads to chromosome aberration, likely explaining blastocyst development failure.

## Discussion

The finite cellular lifespan of primary cells has greatly limited the generation of GMSCs for SCNT. If donor cells can endure in vitro conditions during transgenesis without negatively affecting cell status, this limitation can be overcome. Many researchers have attempted to utilize SCNT embryos derived from immortalized cells for TG modeling. Here, we evaluated SV40LT-mediated immortalized canine and porcine fibroblast cells as nuclei donors for SCNT. A previous study suggested that some malignant characteristics of cancer cells impede the development of SCNT embryos [19]. We showed that SV40LT-expressing fibroblast cells had no transformed cell features, such as anchorage-independent growth in vitro or tumor formation in vivo. Unexpectedly, however, SCNT embryos derived from SV40LT-expressing canine and porcine fibroblast cells showed abnormal embryogenesis. These results suggest that evaluating donor cells based on only transformation characteristics is not appropriate.

Several studies have assessed the feasibility of SCNT and cloning by using immortalized cell lines as nuclei donors [26–28]. Cui et al. and de Semir et al. successfully derived blastocysts from telomerase-mediated immortalized sheep fibroblast cells [26] and SV40LT-mediated transformed rabbit tracheal-epithelial cells, respectively [27]. However, we found that SCNT embryos derived from SV40LT-expressing canine and porcine fibroblast cells failed to develop up to the blastocyst stage. Our results revealed that SV40LT-mediated immortalized canine and porcine fibroblasts have aberrant chromosomal statuses, possibly resulting in disruption of their embryo development. Numerous studies have suggested that blastocyst development is critically affected by the chromosomal status of the embryo [22–25]. Additionally, it has been reported that SV40LT induces chromosomal aberrations or aneuploidy in mammalian diploid cells [29, 30]. One plausible explanation causing this abnormality is that SV40LT directly interacts with and disrupts Bub1 and Bub3, components of the spindle checkpoint complex, leading to chromosomal aberrations. de Semir et al. also showed that chromosomal numbers of SV40-mediated transformed rabbit cells range from 55 to 81 per cell, whereas normal counterpart cells contain 44 chromosomes [27].

In addition, our results showing that the fusion and cleavage rates of SCNT embryos from each donor cell were divergent may be related to the status of donor cells such as their passage number and cell cycle phase [31–33]. However, blastocyst development in all SV40LT groups was remarkably restricted. Embryonic genome activation of porcine and canine embryos occurs in the late 4-cell and later 8-cell stage, respectively [34, 35]. After embryonic genome activation, maternal mRNA derived from the oocyte is degraded and new embryonic transcripts required for development processes begin to be synthesized [36]. Proper functions of de novo developmental factors and epigenetic modifiers are crucial for the next developmental stage [37]. Therefore, SV40LT likely causes aberrant blastocyst development by directly or indirectly inhibiting proper gene regulation, leading to embryogenesis defects and implantation failure. Currently, no developmental genes are known to interact with SV40LT. Therefore, developmental factors affected by SV40LT must be identified by further analysis.

## Conclusions

We evaluated whether a SV40LT-mediated immortalization method was applicable to SCNT. We found that SV40LT is an inappropriate factor for immortalizing nuclear donor cells for SCNT, as it induces a chromosomal aberration and disrupts the embryo developmental process. Therefore, a truncated form of SV40LT enabling extension of the cellular lifespan without affecting embryogenesis or tumorigenic potential should be developed. Additionally, other immortalizing factors or methods for facilitating the establishment of nuclear donor cells for SCNT should be considered.

## Abbreviations

GMSC: Genetically modified somatic cells
H-RAS^V12^: Mutant of human H-RAS substituted 12th glycine to valine
SA-β-gal: Senescence-associated β-galactosidase
SCNT: Somatic cell nuclear transfer
SV40LT: Simian virus 40 large T antigen

## References

[1] Wilmut I, Beaujean N, de Sousa PA, Dinnyes A, King TJ, Paterson LA (2002). Somatic cell nuclear transfer. Nature.

[2] Baguisi A, Behboodi E, Melican DT, Pollock JS, Destrempes MM, Cammuso C (1999). Production of goats by somatic cell nuclear transfer. Nat Biotechnol.

[3] Galli C, Lagutina I, Crotti G, Colleoni S, Turini P, Ponderato N (2003). Pregnancy: a cloned horse born to its dam twin. Nature.

[4] Kato Y, Tani T, Sotomaru Y, Kurokawa K, Kato J, Doguchi H (1998). Eight calves cloned from somatic cells of a single adult. Science.

[5] Lee BC, Kim MK, Jang G, Oh HJ, Yuda F, Kim HJ (2005). Dogs cloned from adult somatic cells. Nature.

[6] Onishi A, Iwamoto M, Akita T, Mikawa S, Takeda K, Awata T (2000). Pig cloning by microinjection of fetal fibroblast nuclei. Science.

[7] Wilmut I, Schnieke AE, McWhir J, Kind AJ, Campbell KH (1997). Viable offspring derived from fetal and adult mammalian cells. Nature.

[8] Yin XJ, Lee HS, Lee YH, Seo YI, Jeon SJ, Choi EG (2005). Cats cloned from fetal and adult somatic cells by nuclear transfer. Reproduction.

[9] Whitelaw CB, Sheets TP, Lillico SG, Telugu BP (2016). Engineering large animal models of human disease. J Pathol.

[10] Kuilman T, Michaloglou C, Mooi WJ, Peeper DS (2010). The essence of senescence. Genes Dev.

[11] Harley CB, Futcher AB, Greider CW (1990). Telomeres shorten during ageing of human fibroblasts. Nature.

[12] Hayflick L (1965). The limited in vitro lifetime of human diploid cell strains. Exp Cell Res.

[13] Ali SH, DeCaprio JA (2001). Cellular transformation by SV40 large T antigen: interaction with host proteins. Semin Cancer Biol.

[14] Ahuja D, Saenz-Robles MT, Pipas JM (2005). SV40 Large T antigen targets multiple cellular pathways to elicit cellular transformation. Oncogene.

[15] Bao L, He L, Chen J, Wu Z, Liao J, Rao L (2011). Reprogramming of ovine adult fibroblasts to pluripotency via drug-inducible expression of defined factors. Cell Res.

[16] Mali P, Ye Z, Hommond HH, Yu X, Lin J, Chen G (2008). Improved efficiency and pace of generating induced pluripotent stem cells from human adult and fetal fibroblasts. Stem Cells.

[17] Gardner HL, Fenger JM, London CA (2016). Dogs as a model for cancer. Annu Rev Anim Biosci.

[18] Jeong YW, Kim JJ, Kim HD, Hwang KC, Hyun SH, Kim NH (2016). Preimplantation development of cloned canine embryos recovered by hysterectomy or surgical uterine flushing and subsequent pregnancy outcomes. Theriogenology.

[19] Hochedlinger K, Blelloch R, Brennan C, Yamada Y, Kim M, Chin L (2004). Reprogramming of a melanoma genome by nuclear transplantation. Genes Dev.

[20] Pipas JM (2009). SV40: Cell transformation and tumorigenesis. Virology.

[21] Cao Z, Sui L, Li Y, Ji S, Zhang X, Zhang Y (2012). Effects of chemically defined medium on early development of porcine embryos derived from parthenogenetic activation and cloning. Zygote.

[22] Alfarawati S, Fragouli E, Colls P, Stevens J, Gutierrez-Mateo C, Schoolcraft WB (2011). The relationship between blastocyst morphology, chromosomal abnormality, and embryo gender. Fertil Steril.

[23] Magli MC, Gianaroli L, Ferraretti AP, Lappi M, Ruberti A, Farfalli V (2007). Embryo morphology and development are dependent on the chromosomal complement. Fertil Steril.

[24] Munné S (2006). Chromosome abnormalities and their relationship to morphology and development of human embryos. Reprod BioMed Online.

[25] Figueira RCS, Setti AS, Braga DP, Iaconelli A, Borges E (2015). Blastocyst morphology holds clues concerning the chromosomal status of the embryo. Int J Fertil Steril.

[26] Cui W, Wylie D, Aslam S, Dinnyes A, King T, Wilmut I (2003). Telomerase-immortalized sheep fibroblasts can be reprogrammed by nuclear transfer to undergo early development. Biol Reprod.

[27] de Semir D, Maurisse R, Du F, Xu J, Yang X, Illek B (2012). Generation of SV40-transformed rabbit tracheal-epithelial-cell-derived blastocyst by somatic cell nuclear transfer. Cell Tissue Res.

[28] Zakhartchenko V, Alberio R, Stojkovic M, Prelle K, Schernthaner W, Stojkovic P (1999). Adult cloning in cattle: potential of nuclei from a permanent cell line and from primary cultures. Mol Reprod Dev.

[29] Ray FA, Peabody DS, Cooper JL, Cram LS, Kraemer PM (1990). SV40 T antigen alone drives karyotype instability that precedes neoplastic transformation of human diploid fibroblasts. J Cell Biochem.

[30] Stewart N, Bacchetti S (1991). Expression of SV40 large T antigen, but not small t antigen, is required for the induction of chromosomal aberrations in transformed human cells. Virology.

[31] Akagi S, Matsukawa K, Takahashi S (2014). Factors affecting the development of somatic cell nuclear transfer embryos in cattle. J Reprod Dev.

[32] Jin YX, Jeon Y, Lee SH, Kwon MS, Kim T, Cui XS (2014). Production of pigs expressing a Transgene under the control of a tetracycline-inducible system. PLoS One.

[33] Zhang Y, Song E, Kim E, Cong P, Lee S, Lee J (2009). Effects of donor cell passage, size and type on development of porcine embryos derived from somatic cell nuclear transfer. Asian-Australas J Anim Sci.

[34] Bysted BV, Greve T (2000). Activation of the embryonic genome in the dog. Theriogenology.

[35] Hyttel P, Laurincik J, Rosenkranz C, Rath D, Niemann H, Ochs RL (2000). Nucleolar proteins and ultrastructure in preimplantation porcine embryos developed in vivo. Biol Reprod.

[36] Prather RS (1993). Nuclear control of early embryonic development in domestic pigs. J Reprod Fertil Suppl.

[37] Cantone I, Fisher AG (2013). Epigenetic programming and reprogramming during development. Nat Struct Mol Biol.

